# Micro-genetic environmental sensitivity across macro-environments of chickens reared in Burkina Faso and France

**DOI:** 10.1186/s12711-023-00854-7

**Published:** 2023-11-30

**Authors:** Mette Dam Madsen, Naomi Duijvesteijn, Julius van der Werf, Sam Clark

**Affiliations:** 1 School of Environmental and Rural Science, University of New England, Armidale, NSW 2351 Australia; 2 Hendrix Genetics, PO Box 114, 5830 AC Boxmeer, The Netherlands

## Abstract

**Background:**

Commercial poultry production systems follow a pyramidal structure with a nucleus of purebred animals under controlled conditions at the top and crossbred animals under commercial production conditions at the bottom. Genetic correlations between the same phenotypes on nucleus and production animals can therefore be influenced by differences both in purebred-crossbred genotypes and in genotype-by-environment interactions across the two environments, known as macro-genetic environmental sensitivity (GES). Within each environment, genotype-by-environment interactions can also occur due to so-called micro-GES. Micro-GES causes heritable variation in phenotypes and decreases uniformity. In this study, genetic variances of body weight (BW) and of micro-GES of BW and the impacts of purebred-crossbred differences and macro-environmental differences on micro-GES of BW were estimated. The dataset contained three subpopulations of slow-growing broiler chickens: purebred chickens (PB) reared in France, and crossbred chickens reared in France (FR) under the same conditions as PB or reared in Burkina Faso (BF) under local conditions. The crossbred chickens were offspring of the same dam line and had PB as their sire line.

**Results:**

Estimates of heritability of BW and micro-GES of BW were 0.54 (SE of 0.02) and 0.06 (0.01), 0.67 (0.03) and 0.03 (0.01), and 0.68 (0.04) and 0.02 (0.01) for the BF, FR, and PB subpopulations, respectively. Estimates of the genetic correlations for BW between the three subpopulations were moderately positive (0.37 to 0.53) and those for micro-GES were weakly to moderately positive (0.01 to 0.44).

**Conclusions:**

The results show that the heritability of the micro-GES of BW varies with macro-environment, which indicates that responses to selection are expected to differ between macro-environments. The weak to moderate positive genetic correlations between subpopulations indicate that both macro-environmental differences and purebred-crossbred differences can cause re-ranking of sires based on their estimated breeding values for micro-GES of BW. Thus, the sire that produces the most variable progeny in one macro-environment may not be the one that produces the most variable offspring in another. Similarly, the sire that produces the most variable purebred progeny may not produce the most variable crossbred progeny. The results highlight the need for investigating micro-GES for all subpopulations included in the selection scheme, to ensure optimal genetic gain in all subpopulations.

**Supplementary Information:**

The online version contains supplementary material available at 10.1186/s12711-023-00854-7.

## Background

Livestock production systems with a pyramidal structure, such as poultry production, rear purebred animals in a bio-secure breeding nucleus at the top of the pyramid, while the crossbred production animals are reared under commercial conditions.

Performance often differs between purebred and crossbred chickens due to differences in their genotypes, which can give rise to purebred-crossbred correlations less than 1. Duenk et al. [1] reported genetic correlations of 0.64 to 0.96 between sires’ estimated breeding values (EBV) for body weight (BW) in purebred offspring versus crossbred offspring when the purebred and crossbred chickens are reared under the same conditions. Genetic correlations less than 1 indicate that re-ranking of sires occurs based on EBV for BW in their purebred offspring versus on EBV for BW in their crossbred offspring. Consequently, the sire with the best purebred offspring performance may not produce the best performing crossbred offspring, and this needs to be considered during selection.

The difference between the environmental conditions at different levels of the breeding pyramid can give rise to genotype-by-environment interactions (G×E) due to differences in macro-genetic environmental sensitivity (GES). Macro-GES refers to genetic differences in sensitivity to definable environments, termed macro-environments [2]. Similar to purebred-crossbred correlations being less than 1, macro-GES can cause the genetic correlation between performance in different macro-environments to be less than 1 [3]. In poultry production, differences in management and biosecurity between the nucleus and production environments are macro-environmental differences, which can give rise to macro-GES. Genetic correlations for BW of purebred chickens reared under commercial conditions versus bio-secure conditions have been reported to range from 0.48 to 0.54 [4]. Similar to purebred-crossbred correlations less than 1, this can affect selection outcomes, as the ranking of sires’ EBV differs between the selection environment and the macro-environments where the offspring are reared.

The structure of the breeding pyramid for chickens implies that both macro-GES and purebred-crossbred differences likely influence traits. Duijvesteijn et al. [5] investigated this by estimating genetic correlations for BW of purebred versus crossbred broilers reared in France under the same conditions, and for these two subpopulations versus a subpopulation of crossbred broilers reared in Burkina Faso under local commercial conditions. The estimate of the purebred-crossbred correlation was considerably higher (0.67) when purebred and crossbred chickens were reared in the same macro-environment, (i.e., France) compared to when the crossbreds were reared in Burkina Faso (0.39). In addition, the estimate of the genetic correlation for crossbreds reared in France versus in Burkina Faso was also higher (0.63) than the purebred-crossbred correlation for BW of purebred chickens reared in France versus crossbred chickens reared in Burkina Faso [5]. These results show that purebred-crossbred differences and macro-GES can amplify the effects of each other when they occur simultaneously, and datasets with specific structures are required to separate the effects of each on genetic correlations.

Within the macro-environment of the different tiers of the breeding pyramid, differences in sensitivity to individual environments (micro-environments) can cause heritable variability of phenotypes [2, 6], which is known as micro-GES. Changing micro-GES through selection can alter the uniformity of production [7, 8] and reducing micro-GES can be beneficial for the population by reducing the sensitivity to transient environmental disturbances in general [9]. Furthermore, reducing micro-GES can be profitable for traits with an intermediate optimum [10] or for traits for which processing phenotypes outside a set range can reduce profits, such as for BW in poultry [9, 11]. For purebred broilers, Mulder et al. [11] reported heritabilities of 0.03 to 0.05 and genetic coefficients of variation from 0.35 to 0.57 for the variability of BW, i.e., the micro-GES of BW, showing that selection on micro-GES should be possible for BW in chickens. Due to the influence of micro-GES on production uniformity, it is relevant to consider micro-GES not only within but also between the different tiers of the breeding pyramid.

Micro-GES of a trait can be influenced by purebred-crossbred differences and by differences in macro-environments. Mulder et al. [12] reported a genetic correlation of micro-GES of eggshell colour of 0.70 between purebred and crossbred chickens reared in different macro-environments. This shows that the sires’ ranking based on EBV for micro-GES of eggshell colour differed when based on phenotypes of purebred chickens reared in one macro-environment versus on phenotypes of crossbred progeny reared in another macro-environment.

The study by Mulder et al. [12] was not designed to evaluate the effects of macro-environments on micro-GES separately from that of purebred-crossbred differences. For chicken breeding programs, it is relevant to estimate the impacts of macro-environments and purebred-crossbred differences on micro-GES of BW separately and together, similar to the study of BW by Duijvesteijn et al. [5]. Such a study will provide information on differences that can be expected in ranking of purebred sires based on EBV for micro-GES of BW for purebred and crossbred offspring, with or without macro-environmental differences, and shed light on which of the two has the largest impact on micro-GES.

The aims of this study were to (1) estimate micro-GES for BW in purebred and crossbred broiler chickens, (2) determine whether micro-GES of BW exhibit macro-GES when phenotypes are measured in distinct macro-environments, and (3) determine if the genetic correlation of micro-GES of BW measured in purebred and crossbred boiler chickens is less than 1 with or without macro-environmental differences.

## Methods

### Data

Live BW records were collected as part of Hendrix Genetics’ Sustainable Access to Poultry Parent Stock to Africa (SAPPSA) project [13]. In the SAPPSA project, a recurrent testing scheme was implemented to improve performance in crossbred dual-purpose chicken under African conditions via selection of pure line males based on crossbred performance. One pure line included in this project is the S-line of the SASSO breeding program used to produce slow-growing broilers and dual-purpose chickens as part of Hendrix Genetics [14]. The S-line is a red-feathered dwarf line used in the C position in the crossing scheme, with the aim to improve egg production while maintaining body weight and improving body conformation.

The dataset contained live BW records from crossbred female progeny from a cross with the S-line as sire line and a single dam line (dam information was sparse). The crossbreds were hatched in France and reared in either Burkina Faso (BF) or France (FR). The BF subpopulation experienced open housing, locally supplied feed, and high temperatures, while the FR subpopulation experienced closed housing, label rouge certified feed, and milder temperatures. The dataset also contained purebred (PB) female offspring from the purebred S-line reared under the same conditions as the FR crossbred females but in a different barn. Thus, differences between the FR crossbreds and the purebred chickens were the result of both macro-environmental differences and purebred-crossbred differences, although the latter are expected to be the primary cause because of the similar management environments. To produce the progeny, sires were mated to either 10 or 18 dams, depending on flock. Dam information was not available on all of the birds. The BF were hatched in 2019–2022, the FR were hatched in 2019–2021, and the PB were hatched in 2017–2021. For BF and FR, all birds were recorded for BW in the same year as they were hatched. For PB, 1554 chickens that hatched in 2021 were recorded in 2022, while the remaining chickens were recorded for BW in the same year as they were hatched.

### Data editing

Location, year, generation, and date of recording were concatenated to form contemporary groups (CG), and phenotypes deviating by more than 3 SD (calculated within CG) from the phenotypic mean of their CG were removed. Records from sires with less than five offspring and CG with offspring from less than five different sires were excluded. Age at measurement was limited to 40 to 55 days. For animals with repeated records measured between 40 and 55 days of age the last record was kept. The final dataset contained records on progeny of 369 sires, 31 of which were represented in all three subpopulations. Summary statistics of the final dataset are in Table 1.

A pedigree of the S-line sires dating three generations back from animals with records (containing 1474 animals) was used to construct the numerator relationship matrix used for analysis.

**Table 1:** Summary statistics for crossbred chickens reared in Burkina Faso (BF) or France (FR), and purebred (PB) chickens

Subpopulation	BF	FR	PB
Records (n)	10,734	6937	3987
Sires with recorded progeny (n)	235	149	215
Contemporary groups (n)	8	3	6
Recording years (year)	2020–22	2020–21	2018–21
Recorded progeny/sire (n)			
Mean	45.68	46.56	18.54
Range	19–59	26–57	5–38
Records/contemporary group (n)			
Mean	1342	2312	665
Range	15–3793	1786–3218	429–787
Sires per contemporary group (n)			
Mean	60.75	74.67	35.83
Range	13–82	74–75	34–41
Body weight (g)			
Mean	573.0	660.4	639.6
SD	119.6	87.9	117.4
Range	270–1015	395–923	350–1030

### Statistical analyses

A multivariate model was fitted to estimate the variance components of BW and of micro-GES of BW. This model treated the phenotype from each subpopulation as different traits and included a double hierarchical generalised linear model (DHGLM) on BW from each subpopulation.

A DHGLM consists of two interdependent parts, a mean part and a dispersion part, fitted as a bivariate model for a single measured trait. The mean part uses the measured phenotype as response variable, while the dispersion part uses a calculated phenotype based on the residuals (or dispersion) of the measured phenotypes (see Eq. (5)) [15]. Thus, the multivariate model for BW and micro-GES of BW in the three subpopulations included six traits and allowed for the simultaneous estimation of all variance components, including covariances between subpopulations. The multivariate model was fitted as a sire model, because dam information was too sparse to construct a full pedigree on all birds and DHGLM fitted on the individual level require repeated measures on all animals to obtain accurate estimates [16].

The model was:

$$\left[\begin{array}{c}\begin{array}{c}{\mathbf{y}}_{\mathbf{BF}}\\ {{\mathbf{y}}_{\mathbf{d}}}_{\mathbf{BF}}\\ {\mathbf{y}}_{\mathbf{FR}}\end{array}\\ \begin{array}{c}{{\mathbf{y}}_{\mathbf{d}}}_{\mathbf{FR}}\\ {\mathbf{y}}_{\mathbf{PB}}\\ {{\mathbf{y}}_{\mathbf{d}}}_{\mathbf{PB}}\end{array}\end{array}\right]=\left[\begin{array}{cc}\begin{array}{ccc}\begin{array}{c}\begin{array}{c}{\mathbf{X}}_{\mathbf{BF}}\\ {\mathbf{0}}\\ {\mathbf{0}}\end{array}\\ \begin{array}{c}{\mathbf{0}}\\ {\mathbf{0}}\\ {\mathbf{0}}\end{array}\end{array}& \begin{array}{c}\begin{array}{c}{\mathbf{0}}\\ {{\mathbf{X}}_{\mathbf{d}}}_{\mathbf{BF}}\\ {\mathbf{0}}\end{array}\\ \begin{array}{c}{\mathbf{0}}\\ {\mathbf{0}}\\ {\mathbf{0}}\end{array}\end{array}& \begin{array}{c}\begin{array}{c}{\mathbf{0}}\\ {\mathbf{0}}\\ {\mathbf{X}}_{\mathbf{FR}}\end{array}\\ \begin{array}{c}{\mathbf{0}}\\ {\mathbf{0}}\\ {\mathbf{0}}\end{array}\end{array}\end{array}& \begin{array}{ccc}\begin{array}{c}\begin{array}{c}{\mathbf{0}}\\ {\mathbf{0}}\\ {\mathbf{0}}\end{array}\\ \begin{array}{c}{{\mathbf{X}}_{\mathbf{d}}}_{\mathbf{FR}}\\ {\mathbf{0}}\\ {\mathbf{0}}\end{array}\end{array}& \begin{array}{c}\begin{array}{c}{\mathbf{0}}\\ {\mathbf{0}}\\ {\mathbf{0}}\end{array}\\ \begin{array}{c}{\mathbf{0}}\\ {\mathbf{X}}_{\mathbf{PB}}\\ {\mathbf{0}}\end{array}\end{array}& \begin{array}{c}\begin{array}{c}{\mathbf{0}}\\ {\mathbf{0}}\\ {\mathbf{0}}\end{array}\\ \begin{array}{c}{\mathbf{0}}\\ {\mathbf{0}}\\ {{\mathbf{X}}_{\mathbf{d}}}_{\mathbf{PB}}\end{array}\end{array}\end{array}\end{array}\right]\left[\begin{array}{c}\begin{array}{c}{\mathbf{b}}_{\mathbf{BF}}\\ {{\mathbf{b}}_{\mathbf{d}}}_{\mathbf{BF}}\\ {\mathbf{b}}_{\mathbf{FR}}\end{array}\\ \begin{array}{c}{{\mathbf{b}}_{\mathbf{d}}}_{\mathbf{FR}}\\ {\mathbf{b}}_{\mathbf{PB}}\\ {{\mathbf{b}}_{\mathbf{d}}}_{\mathbf{PB}}\end{array}\end{array}\right]+\left[\begin{array}{cc}\begin{array}{ccc}\begin{array}{c}\begin{array}{c}{\mathbf{Z}}_{\mathbf{BF}}\\ {\mathbf{0}}\\ {\mathbf{0}}\end{array}\\ \begin{array}{c}{\mathbf{0}}\\ {\mathbf{0}}\\ {\mathbf{0}}\end{array}\end{array}& \begin{array}{c}\begin{array}{c}{\mathbf{0}}\\ {{\mathbf{Z}}_{\mathbf{d}}}_{\mathbf{BF}}\\ {\mathbf{0}}\end{array}\\ \begin{array}{c}{\mathbf{0}}\\ {\mathbf{0}}\\ {\mathbf{0}}\end{array}\end{array}& \begin{array}{c}\begin{array}{c}{\mathbf{0}}\\ {\mathbf{0}}\\ {\mathbf{Z}}_{\mathbf{FR}}\end{array}\\ \begin{array}{c}{\mathbf{0}}\\ {\mathbf{0}}\\ {\mathbf{0}}\end{array}\end{array}\end{array}& \begin{array}{ccc}\begin{array}{c}\begin{array}{c}{\mathbf{0}}\\ {\mathbf{0}}\\ {\mathbf{0}}\end{array}\\ \begin{array}{c}{{\mathbf{Z}}_{\mathbf{d}}}_{\mathbf{FR}}\\ {\mathbf{0}}\\ {\mathbf{0}}\end{array}\end{array}& \begin{array}{c}\begin{array}{c}{\mathbf{0}}\\ {\mathbf{0}}\\ {\mathbf{0}}\end{array}\\ \begin{array}{c}{\mathbf{0}}\\ {\mathbf{Z}}_{\mathbf{PB}}\\ {\mathbf{0}}\end{array}\end{array}& \begin{array}{c}\begin{array}{c}{\mathbf{0}}\\ {\mathbf{0}}\\ {\mathbf{0}}\end{array}\\ \begin{array}{c}{\mathbf{0}}\\ {\mathbf{0}}\\ {{\mathbf{Z}}_{\mathbf{d}}}_{\mathbf{PB}}\end{array}\end{array}\end{array}\end{array}\right]\left[\begin{array}{c}\begin{array}{c}{\mathbf{s}}_{\mathbf{BF}}\\ {{\mathbf{s}}_{\mathbf{d}}}_{\mathbf{BF}}\\ {\mathbf{s}}_{\mathbf{FR}}\end{array}\\ \begin{array}{c}{{\mathbf{s}}_{\mathbf{d}}}_{\mathbf{FR}}\\ {\mathbf{s}}_{\mathbf{PB}}\\ {{\mathbf{s}}_{\mathbf{d}}}_{\mathbf{PB}}\end{array}\end{array}\right]+\left[\begin{array}{c}\begin{array}{c}{\mathbf{es}}_{\mathbf{BF}}\\ {{\mathbf{es}}_{\mathbf{d}}}_{\mathbf{BF}}\\ {\mathbf{es}}_{\mathbf{FR}}\end{array}\\ \begin{array}{c}{{\mathbf{es}}_{\mathbf{d}}}_{\mathbf{FR}}\\ {\mathbf{es}}_{\mathbf{PB}}\\ {{\mathbf{es}}_{\mathbf{d}}}_{\mathbf{PB}}\end{array}\end{array}\right],$$

where ${\mathbf{y}}_{j}$ is the vector of the measured phenotypes (BW) for the mean part of the DHGLM for subpopulation $j$ $(j\in BF, FR, PB)$, and ${{\mathbf{y}}_{\mathbf{d}}}_{j}$ is the vector of the calculated dispersion phenotype (see Eq. (5)) for subpopulation $j$. ${\mathbf{b}}_{j}$ and ${{\mathbf{b}}_{\mathbf{d}}}_{j}$ are the vectors of the fixed effect of CG and the fixed regression on age at measurement for the observed and the dispersion phenotypes, respectively, for subpopulation $j$. ${\mathbf{s}}_{j}$ is the vector of random genetic sire effects of the observed phenotypes for subpopulation $j$, ${{\mathbf{s}}_{\mathbf{d}}}_{j}$ is the vector of the random genetic sire effects of the dispersion phenotypes for subpopulation $j$. ${\mathbf{es}}_{j}$ and ${\mathbf{es}}_{{\mathbf{d}}_{\varvec{j}}}$ are the vectors of residuals of the observed and dispersion phenotypes, respectively, for subpopulation $j$. Matrices ${\mathbf{X}}_{j}$, ${{\mathbf{X}}_{\mathbf{d}}}_{j}$, ${\mathbf{Z}}_{j}$, and ${{\mathbf{Z}}_{\mathbf{d}}}_{j}$ are design matrices corresponding to ${\mathbf{b}}_{j}$, ${{\mathbf{b}}_{\mathbf{d}}}_{j}$, ${\mathbf{s}}_{j}$, and ${{\mathbf{s}}_{\mathbf{d}}}_{j}$, respectively.

The distributional assumptions for the random genetic sire effects were:

$$\left[\begin{array}{c}\begin{array}{c}{\mathbf{s}}_{\mathbf{BF}}\\ {{\mathbf{s}}_{\mathbf{d}}}_{\mathbf{BF}}\\ {\mathbf{s}}_{\mathbf{FR}}\end{array}\\ \begin{array}{c}{{\mathbf{s}}_{\mathbf{d}}}_{\mathbf{FR}}\\ {\mathbf{s}}_{\mathbf{PB}}\\ {{\mathbf{s}}_{\mathbf{d}}}_{\mathbf{PB}}\end{array}\end{array}\right]\sim\text{MVN}\left(\mathbf{0},\left[\begin{array}{cc}\begin{array}{ccc}\begin{array}{c}\begin{array}{c}{{\upsigma }}_{{\text{s}}_{\text{BF}}}^{2}\\ {{\upsigma }}_{{{\text{s}}_{\text{d}}}_{\text{BF}},{\text{s}}_{\text{BF}}}\\ {{\upsigma }}_{{\text{s}}_{\text{FR}},{\text{s}}_{\text{BF}}}\end{array}\\ \begin{array}{c}{{\upsigma }}_{{{\text{s}}_{\text{d}}}_{\text{FR}},{\text{s}}_{\text{BF}}}\\ {{\upsigma }}_{{\text{s}}_{\text{PB}},{\text{s}}_{\text{BF}}}\\ {{\upsigma }}_{{{\text{s}}_{\text{d}}}_{\text{PB}},{\text{s}}_{\text{BF}}}\end{array}\end{array}& \begin{array}{c}\begin{array}{c}{{\upsigma }}_{{\text{s}}_{\text{BF}},{{\text{s}}_{\text{d}}}_{\text{BF}}}\\ {{\upsigma }}_{{{\text{s}}_{\text{d}}}_{\text{BF}}}^{2}\\ {{\upsigma }}_{{\text{s}}_{\text{FR}},{{\text{s}}_{\text{d}}}_{\text{BF}}}\end{array}\\ \begin{array}{c}{{\upsigma }}_{{{\text{s}}_{\text{d}}}_{\text{FR}},{{\text{s}}_{\text{d}}}_{\text{BF}}}\\ {{\upsigma }}_{{\text{s}}_{\text{PB}},{{\text{s}}_{\text{d}}}_{\text{BF}}}\\ {{\upsigma }}_{{{\text{s}}_{\text{d}}}_{\text{PB}},{{\text{s}}_{\text{d}}}_{\text{BF}}}\end{array}\end{array}& \begin{array}{c}\begin{array}{c}{{\upsigma }}_{{\text{s}}_{\text{BF}},{\text{s}}_{\text{FR}}}\\ {{\upsigma }}_{{{\text{s}}_{\text{d}}}_{\text{BF}},{\text{s}}_{\text{FR}}}\\ {{\upsigma }}_{{\text{s}}_{\text{FR}}}^{2}\end{array}\\ \begin{array}{c}{{\upsigma }}_{{{\text{s}}_{\text{d}}}_{\text{FR}},{\text{s}}_{\text{FR}}}\\ {{\upsigma }}_{{\text{s}}_{\text{PB}},{\text{s}}_{\text{FR}}}\\ {{\upsigma }}_{{{\text{s}}_{\text{d}}}_{\text{PB}},{\text{s}}_{\text{FR}}}\end{array}\end{array}\end{array}& \begin{array}{ccc}\begin{array}{c}\begin{array}{c}{{\upsigma }}_{{\text{s}}_{\text{BF}},{{\text{s}}_{\text{d}}}_{\text{FR}}}\\ {{\upsigma }}_{{{\text{s}}_{\text{d}}}_{\text{BF}},{{\text{s}}_{\text{d}}}_{\text{FR}}}\\ {{\upsigma }}_{{\text{s}}_{\text{FR}},{{\text{s}}_{\text{d}}}_{\text{FR}}}\end{array}\\ \begin{array}{c}{{\upsigma }}_{{{\text{s}}_{\text{d}}}_{\text{FR}}}^{2}\\ {{\upsigma }}_{{\text{s}}_{\text{PB}},{{\text{s}}_{\text{d}}}_{\text{FR}}}\\ {{\upsigma }}_{{{\text{s}}_{\text{d}}}_{\text{PB}},{{\text{s}}_{\text{d}}}_{\text{FR}}}\end{array}\end{array}& \begin{array}{c}\begin{array}{c}{{\upsigma }}_{{\text{s}}_{\text{BF}},{\text{s}}_{\text{PB}}}\\ {{\upsigma }}_{{{\text{s}}_{\text{d}}}_{\text{BF}},{\text{s}}_{\text{PB}}}\\ {{\upsigma }}_{{\text{s}}_{\text{FR}},{\text{s}}_{\text{PB}}}\end{array}\\ \begin{array}{c}{{\upsigma }}_{{{\text{s}}_{\text{d}}}_{\text{FR}},{\text{s}}_{\text{PB}}}\\ {{\upsigma }}_{{\text{s}}_{\text{PB}}}^{2}\\ {{\upsigma }}_{{{\text{s}}_{\text{d}}}_{\text{PB}},{\text{s}}_{\text{PB}}}\end{array}\end{array}& \begin{array}{c}\begin{array}{c}{{\upsigma }}_{{\text{s}}_{\text{BF}},{{\text{s}}_{\text{d}}}_{\text{PB}}}\\ {{\upsigma }}_{{{\text{s}}_{\text{d}}}_{\text{BF}},{{\text{s}}_{\text{d}}}_{\text{PB}}}\\ {{\upsigma }}_{{\text{s}}_{\text{FR}},{{\text{s}}_{\text{d}}}_{\text{PB}}}\end{array}\\ \begin{array}{c}{{\upsigma }}_{{{\text{s}}_{\text{d}}}_{\text{FR}}{{\text{s}}_{\text{d}}}_{\text{PB}}}\\ {{\upsigma }}_{{\text{s}}_{\text{PB}},{{\text{s}}_{\text{d}}}_{\text{PB}}}\\ {{\upsigma }}_{{{\text{s}}_{\text{d}}}_{\text{PB}}}^{2}\end{array}\end{array}\end{array}\end{array}\right]\otimes \mathbf{A}\right),$$

where MVN is multi-variate normal distribution, $\mathbf{A}$ is the numerator relationship matrix based on the sire pedigree, and $\otimes$ is the Kronecker product. The distribution assumptions for the residuals were:

$$\left[\begin{array}{c}\begin{array}{c}{\mathbf{es}}_{\mathbf{BF}}\\ {{\mathbf{es}}_{\mathbf{d}}}_{\mathbf{BF}}\\ {\mathbf{es}}_{\mathbf{FR}}\end{array}\\ \begin{array}{c}{{\mathbf{es}}_{\mathbf{d}}}_{\mathbf{FR}}\\ {\mathbf{es}}_{\mathbf{PB}}\\ {{\mathbf{es}}_{\mathbf{d}}}_{\mathbf{PB}}\end{array}\end{array}\right]\sim\text{MVN}\left({\mathbf{0}},\left[\begin{array}{cc}\begin{array}{ccc}\begin{array}{c}\begin{array}{c}{\mathbf{W}}_{\mathbf{BF}}^{-1}{{\upsigma }}_{{\in }_{\text{BF}}}^{2}\\ {\mathbf{0}}\\ {\mathbf{0}}\end{array}\\ \begin{array}{c}{\mathbf{0}}\\ {\mathbf{0}}\\ {\mathbf{0}}\end{array}\end{array}& \begin{array}{c}\begin{array}{c}{\mathbf{0}}\\ {\mathbf{W}}_{{\mathbf{d}}_{\mathbf{BF}}}^{-1}{{\upsigma }}_{{\in }_{{\text{d}}_{\text{BF}}}}^{2}\\ {\mathbf{0}}\end{array}\\ \begin{array}{c}{\mathbf{0}}\\ {\mathbf{0}}\\ {\mathbf{0}}\end{array}\end{array}& \begin{array}{c}\begin{array}{c}{\mathbf{0}}\\ {\mathbf{0}}\\ {\mathbf{W}}_{\mathbf{FR}}^{-1}{{\upsigma }}_{{\in }_{\text{FR}}}^{2}\end{array}\\ \begin{array}{c}{\mathbf{0}}\\ {\mathbf{0}}\\ {\mathbf{0}}\end{array}\end{array}\end{array}& \begin{array}{ccc}\begin{array}{c}\begin{array}{c}{\mathbf{0}}\\ {\mathbf{0}}\\ {\mathbf{0}}\end{array}\\ \begin{array}{c}{\mathbf{W}}_{{\mathbf{d}}_{\mathbf{FR}}}^{-1}{{\upsigma }}_{{\in }_{{\text{d}}_{\text{FR}}}}^{2}\\ {\mathbf{0}}\\ {\mathbf{0}}\end{array}\end{array}& \begin{array}{c}\begin{array}{c}{\mathbf{0}}\\ {\mathbf{0}}\\ {\mathbf{0}}\end{array}\\ \begin{array}{c}{\mathbf{0}}\\ {\mathbf{W}}_{\mathbf{PB}}^{-1}{{\upsigma }}_{{\in }_{\text{PB}}}^{2}\\ {\mathbf{0}}\end{array}\end{array}& \begin{array}{c}\begin{array}{c}{\mathbf{0}}\\ {\mathbf{0}}\\ {\mathbf{0}}\end{array}\\ \begin{array}{c}{\mathbf{0}}\\ {\mathbf{0}}\\ {\mathbf{W}}_{{\mathbf{d}}_{\mathbf{PB}}}^{-1}{{\upsigma }}_{{\in }_{{\text{d}}_{\text{PB}}}}^{2}\end{array}\end{array}\end{array}\end{array}\right]\right),$$

where ${\mathbf{W}}_{j}$ and ${\mathbf{W}}_{{\mathbf{d}}_{j}}$ are weights for the residual variances for ${\mathbf{y}}_{j}$ and ${{\mathbf{y}}_{\mathbf{d}}}_{j}$, respectively, for subpopulation $j$, with:

1 $${\mathbf{W}}_{j}=\text{diag}{\left({\widehat{{\mathbf{y}}_{\mathbf{d}}}}_{j}\right)}^{-1},$$

2 $${\mathbf{W}}_{{\mathbf{d}}_{j}}=\frac{1-{\mathbf{h}}_{j}}{2},$$

where ${\widehat{{\mathbf{y}}_{\mathbf{d}}}}_{j}$ is the vector of predicted dispersion phenotypes in subpopulation $j$ and ${\mathbf{h}}_{j}$ the vector of the diagonal elements of the hat-matrix of ${\mathbf{y}}_{j}$ $(\widehat{{\mathbf{y}}_{j}}={\mathbf{H}}_{j}{\mathbf{y}}_{j})$, also known as the leverage [17]. Because the weights already contain the reciprocal of the residual variance per observation, ${{\upsigma }}_{{\in }_{j}}^{2}$ and ${{\upsigma }}_{{\in }_{{\text{d}}_{j}}}^{2}$ are expected to be equal to 1. However estimating these variances allows for more flexibility in the model [16]. The residual variances (${{\upsigma }}_{{\text{e}\text{s}}_{j}}^{2}$ and ${{\upsigma }}_{{\text{e}\text{s}}_{{\text{d}}_{j}}}^{2}$) were then calculated as:

3 $${{\upsigma }}_{{\text{es}}_{j}}^{2}=\frac{{{\upsigma }}_{{\in }_{j}}^{2}}{\left(\frac{\text{tr}\left({\mathbf{W}}_{j}\right)}{{\text{n}}_{j}}\right)},$$

4 $${{\upsigma }}_{{\text{es}}_{{\text{d}}_{j}}}^{2}=\frac{{{\upsigma }}_{{\in }_{{\text{d}}_{j}}}^{2}}{\left(\frac{\text{tr}\left({\mathbf{W}}_{{\mathbf{d}}_{j}}\right)}{{\text{n}}_{j}}\right)},$$

where ${\text{n}}_{j}$ is the number of records for subpopulation $j$.

The phenotypes used to estimate micro-GES $({{\mathbf{y}}_{\mathbf{d}}}_{j})$ were based on the squared residuals of ${\mathbf{y}}_{j}$, calculated as:

5 $${{\text{y}}_{{{\text{d}}_{{j_i}}}}} = \frac{{{\widehat {es}}_{{j_i}}^{2}}}{{1 - {{\text{h}}_{{j_i}}}}}$$

where ${{\widehat{es}}_{{j_i}}^{2}}$ is the squared estimated residual of observation $i$ for subpopulation $j$ and ${{\text{h}}_{j}}_{i}$ is the leverage of observation $i$ for subpopulation $j$ [15].

The multivariate model was fitted using the DMU software package [18],with the following algorithm:

1. Initialize the model by running a univariate model on ${\mathbf{y}}_{\mathbf{BF}}$, ${\mathbf{y}}_{\mathbf{FR}}$, and ${\mathbf{y}}_{\mathbf{PB}}$ with homogeneous residual variances.
2. Calculate ${{\mathbf{y}}_{\mathbf{d}}}_{\mathbf{BF}}$, ${{\mathbf{y}}_{\mathbf{d}}}_{\mathbf{FR}}$, and ${{\mathbf{y}}_{\mathbf{d}}}_{\mathbf{PB}}$ using Eq. (5) and ${\mathbf{W}}_{{\mathbf{d}}_{\mathbf{BF}}}$, ${\mathbf{W}}_{{\mathbf{d}}_{\mathbf{FR}}}$, and ${\mathbf{W}}_{{\mathbf{d}}_{\mathbf{PB}}}$ using Eq. (2) based on ${\widehat{\mathbf{es}}}_{\mathbf{BF}}$, ${\widehat{\mathbf{es}}}_{\mathbf{FR}}$, ${\widehat{\mathbf{es}}}_{\mathbf{PB}}$, ${\mathbf{h}}_{\mathbf{BF}}$, ${\mathbf{h}}_{\mathbf{FR}}$, and ${\mathbf{h}}_{\mathbf{PB}}$, as estimated in step 1.
3. Run a univariate generalized linear mixed model with a log-link function on ${{\mathbf{y}}_{\mathbf{d}}}_{\mathbf{BF}}$, ${{\mathbf{y}}_{\mathbf{d}}}_{\mathbf{FR}}$, and ${{\mathbf{y}}_{\mathbf{d}}}_{\mathbf{PB}}$.
4. Calculate ${\mathbf{W}}_{\mathbf{BF}}$, ${\mathbf{W}}_{\mathbf{FR}}$, and ${\mathbf{W}}_{\mathbf{PB}}$ using Eq. (1) based on $\widehat{{{\mathbf{y}}_{\mathbf{d}}}_{\mathbf{BF}}}$, $\widehat{{{\mathbf{y}}_{\mathbf{d}}}_{\mathbf{FR}}}$, and $\widehat{{{\mathbf{y}}_{\mathbf{d}}}_{\mathbf{PB}}}$, as estimated in step 3.
5. Run the multivariate model.
6. Update the ${{\mathbf{y}}_{\mathbf{d}}}_{\mathbf{BF}}$, ${{\mathbf{y}}_{\mathbf{d}}}_{\mathbf{FR}}$, ${{\mathbf{y}}_{\mathbf{d}}}_{\mathbf{PB}}$, ${\mathbf{W}}_{{\mathbf{d}}_{\mathbf{BF}}}$, ${\mathbf{W}}_{{\mathbf{d}}_{\mathbf{FR}}}$, ${\mathbf{W}}_{{\mathbf{d}}_{\mathbf{PB}}}$, ${\mathbf{W}}_{\mathbf{BF}}$, ${\mathbf{W}}_{\mathbf{FR}}$, and ${\mathbf{W}}_{\mathbf{PB}}$ based on $\widehat{{\mathbf{e}\mathbf{s}}_{\mathbf{BF}}}$, $\widehat{{\mathbf{e}\mathbf{s}}_{\mathbf{FR}}}$, $\widehat{{\mathbf{e}\mathbf{s}}_{\mathbf{PB}}}$, ${\mathbf{h}}_{\mathbf{BF}}$, ${\mathbf{h}}_{\mathbf{FR}}$, ${\mathbf{h}}_{\mathbf{P}\mathbf{B}}$, $\widehat{{{\mathbf{y}}_{\mathbf{d}}}_{\mathbf{BF}}}$, $\widehat{{{\mathbf{y}}_{\mathbf{d}}}_{\mathbf{FR}}}$, and $\widehat{{{\mathbf{y}}_{\mathbf{d}}}_{\mathbf{PB}}}$, as estimated in step 5.

Iterate steps 5 and 6 until convergence, which is considered to occur when the difference between estimated (co-)variances from run $t$ and run $t-1$ is lower than 10^−6^.

### Post-analysis corrections and heritabilities

As the model was implemented as a sire model, the additive genetic sire variance $({\sigma }_{s}^{2})$ was estimated rather than the additive genetic variance $({\sigma }_{a}^{2})$. The additive genetic sire variance is only ¼${\sigma}_{a}^{2}$ and the remaining ¾${\sigma}_{a}^{2}$ is included in the residual variance. For the observed phenotype for subpopulation $j$, the additive genetic was computed as:

6 $${{\upsigma }}_{{\text{a}}_{j}}^{2}=4{{\upsigma }}_{{\text{s}}_{j}}^{2},$$

and the heritability of the observed phenotype for subpopulation $j\;({\text{h}}_{j}^{2})$ was calculated as:

7 $${\text{h}}_{j}^{2}=\frac{4{{\upsigma }}_{{\text{s}}_{j}}^{2}}{{{\upsigma }}_{{\text{s}}_{j}}^{2}+{{\upsigma }}_{{\text{es}}_{j}}^{2}}.$$

For the dispersion part of the models, computation of the additive genetic variance had to consider the fact that ¾ of the additive genetic variance was included in the residual variance of the mean part of the model, thus contributing to the calculated dispersion phenotype, and was calculated for subpopulation *j* following Madsen et al. [19] as:

8 $${{\upsigma }}_{{{\text{a}}_{\text{d}}}_{j}}^{2}={4{{\upsigma }}_{{{\text{s}}_{\text{d}}}_{j}}^{2}\left(\frac{{{\upsigma }}_{{\text{e}\text{s}}_{j}}^{2}}{{{\upsigma }}_{{\text{es}}_{j}}^{2}-3{{\upsigma }}_{{\text{s}}_{j}}^{2}}\right)}^{2},$$

and the heritability of the dispersion of subpopulation $j$ on the log-scale $({\text{h}}_{{j}_{d}}^{2})$ was calculated as:

9 $${\text{h}}_{{\text{d}}_{j}}^{2}=\frac{{{\upsigma }}_{{{\text{a}}_{\text{d}}}_{j}}^{2}}{\frac{1}{4}{{\upsigma }}_{{{\text{a}}_{\text{d}}}_{j}}^{2}+{{\upsigma }}_{{{\text{es}}_{\text{d}}}_{j}}^{2}}.$$

As the model used a log-link function to fit the dispersion, the estimated variances of the mean and dispersion were on different scales. Mulder et al. [11] showed how to convert the estimated additive genetic variance of the dispersion from the log-scale to the scale of measurement. However, the additive genetic variance of the dispersion on the measurement scale is typically very large compared to the additive genetic variance of the direct genetic effects, because the dispersion part of the DHGLM estimates the genetic variance of the squared residuals of the observed phenotype. In this study, BW was measured in g and the calculated dispersion phenotype then had the units of ${\text{g}}^{2}$. Consequently, the unit of the additive genetic variance of the dispersion phenotypes on the measurement scale was ${\text{g}}^{4}$. To improve the comparison between the additive genetic variance of BW $({\text{g}}^{2})$ and the additive genetic variance of micro-GES of BW (g^4^), the additive genetic standard deviation on the measurement scale (${\text{g}}^{2}$,), was reported as:

10 $${{\upsigma }}_{{{\text{a}}_{\text{d}}}_{j}}^{{*}}=\sqrt{{{\upsigma }}_{{{\text{a}}_{\text{d}}}_{j}}^{{2}^{{*}}}}=\sqrt{2{\left({{\upsigma }}_{{\text{e}\text{s}}_{j}}^{2}-3{{\upsigma }}_{{\text{s}}_{j}}^{2}\right)}^{2}{\text{h}}_{{\text{d}}_{j}}^{2}}.$$

The heritability on the scale of measurement $({\text{h}}_{d}^{{2}^{*}})$ was calculated following Mulder et al. [20]:

11 $${\text{h}}_{{\text{d}}_{j}}^{{2}^{{*}}}=\frac{{{\upsigma }}_{{{\text{a}}_{\text{d}}}_{j}}^{{2}^{{*}}}}{2{\left({{\upsigma }}_{{\text{a}}_{j}}^{2}+{{\upsigma }}_{{\text{es}}_{j}}^{2}-3{{\upsigma }}_{{\text{s}}_{j}}^{2}\right)}^{2}+3{{\upsigma }}_{{{\text{a}}_{\text{d}}}_{j}}^{{2}^{{*}}}}.$$

The genetic coefficient of variation of the dispersion $({\text{GCV}}_{\text{d}})$ was calculated as a measure of the size of the possible selection response relative to the current residual variance [11]. The genetic coefficient of variation for the dispersion for subpopulation $j$ was calculated following Mulder et al. [11] as:

12 $${{\text{GCV}}_{\text{d}}}_{j}=\frac{{{\upsigma }}_{{{\text{a}}_{\text{d}}}_{j}}^{{*}}}{{{\upsigma }}_{{\text{ea}}_{j}}^{2}}.$$

The EBV were converted from the sire effect to the animal effect level by:

13 $${\text{a}}_{j}=2{\text{s}}_{j},$$

for the observed phenotypes of the model, while the EBV of the dispersion phenotypes (i.e., micro-GES) were corrected as described in Madsen et al. [19]:

14 $${{\text{a}}_{\text{d}}}_{j}=2{{\text{s}}_{\text{d}}}_{j}\left(\frac{{{\upsigma }}_{{\text{es}}_{j}}^{2}}{{{\upsigma }}_{{\text{es}}_{j}}^{2}-3{{\upsigma }}_{{\text{s}}_{j}}^{2}}\right).$$

To facilitate comparison across subpopulations, EBV were standardised by dividing them by the genetic standard deviation of the trait within the subpopulation:

15 $${{\text{a}}_{j}}_{sd}=\frac{{\text{a}}_{j}}{{{\upsigma }}_{{\text{a}}_{j}}},$$

16 $${{{\text{a}}_{\text{d}}}_{j}}_{sd}=\frac{{{\text{a}}_{\text{d}}}_{j}}{{{\upsigma }}_{{{\text{a}}_{d}}_{j}}}.$$

## Results

### Additive genetic variances and heritabilities

Estimates of heritability for BW were high for all subpopulations (Table 2). Different levels of genetic variance were observed between the three subpopulations, with the BF and FR subpopulations having, respectively, 28 and 18% less additive genetic variance than the PB subpopulation. The FR and PB subpopulations had similar heritabilities, while BF had 21 and 19% lower heritability than PB and FR, respectively.

Non-zero genetic variances of the dispersion were estimated for the BF and FR subpopulations. Heritability estimates of micro-GES of BW on the log-scale were moderate for all three subpopulations, but for the PB subpopulation it was not significantly different from zero. On the log- and measurement scale, heritability estimates for the dispersion were low but significant for all subpopulations.

Estimates of heritability and of the genetic coefficient of variation of the dispersion of BW were 1.1 to 2.5 times larger for the BF than for the FR and PB subpopulations and at most 1.1 times larger for the FR than the PB subpopulation.

**Table 2:** Additive genetic variance and heritabilities (standard errors) in crossbred chicken reared in Burkina Faso (BF) or France (FR) and purebred (PB) chickens reared in France

Subpopulation	BF	FR	PB
${{\sigma }}_{{a}}^{2}$	2321 (271)	2637 (356)	3226 (464)
${{\sigma }}_{{{a}}_{{d}}}^{2}$	1.02 (0.28)	0.67 (0.39)	0.57 (0.41)
${{\sigma }}_{{{a}}_{{d}}}^{{*}}$	1561 (111)	917 (122)	1042 (229)
${{\sigma }}_{{e}}^{2}$	1942 (211)	1279 (274)	1526 (366)
${{\sigma }}_{{{e}}_{{d}}}^{2}$	1.93 (0.05)	1.87 (0.04)	1.80 (0.06)
${{h}}^{2}$	0.54 (0.06)	0.67 (0.08)	0.68 (0.08)
${{h}}_{{d}}^{2}$	0.32 (0.06)	0.26 (0.11)	0.23 (0.13)
${{h}}_{{d}}^{{2}^{{*}}}$	0.06 (0.01)	0.03 (0.01)	0.02 (0.01)
${GCV}$	0.08 (0.00)	0.08 (0.01)	0.09 (0.01)
${{GCV}}_{{d}}$	0.80 (0.08)	0.72 (0.15)	0.68 (0.19)

${\sigma }_{a}^{2}$, ${\sigma }_{e}^{2}$ and ${h}^{2}$ = additive genetic variance, residual variance and heritability, respectively, of BW. ${\sigma }_{{a}_{d}}^{2}$, ${\sigma }_{{e}_{d}}^{2}$ and ${h}_{d}^{2}$ = additive genetic variance, residual variance and heritability, respectively, of micro-GES of BW on the log-scale. ${\sigma }_{{a}_{d}}^{*}$, ${h}_{d}^{{2}^{{*}}}$ and ${GCV}_{d}$ = additive genetic SD, heritability and genetic coefficient of variation of micro-GES of BW on the measurement scale

### Genetic correlations within and across subpopulations

Three types of genetic correlations were estimated.

The first type was the genetic correlation between BW and micro-GES of BW within subpopulation. For these genetic correlations, positive estimates indicate that greater BW was associated with greater micro-GES. Estimates of the genetic correlation between BW and micro-GES of BW in the BF and PB subpopulations were weakly negative, while it was moderately positive for the FR subpopulation (Table 3).

The second type was the genetic correlation for BW or micro-GES of BW between subpopulations. These correlations indicate the degree of re-ranking of EBV across subpopulations, with estimates of 1 and − 1 indicating no re-ranking and complete inversion of the ranking, respectively. All genetic correlations for BW between the different subpopulations were estimated to be moderately positive. The estimate of the genetic correlation for micro-GES of BW between subpopulations was moderately positive between the BF and FR and between the BF and PB subpopulations, but low between the FR and PB subpopulations. The genetic correlation between micro-GES across subpopulations had large SE.

The third type was the genetic correlation between BW in one subpopulation and micro-GES of BW in another subpopulation. Estimates of these genetic correlations were weakly to moderately positive with large SE.

**Table 3:** Estimates of genetic correlations between body weight (BW) and micro-genetic environmental sensitivity (GES) of BW in crossbred chickens reared in Burkina Faso (BF) or France (FR) and purebred (PB) chickens reared in France

Subpopulation		BF		FR		PB
		BW	Micro-GES	BW	Micro-GES	BW
BF	Micro-GES	− 0.14 (0.10)				
FR	BW	0.43 (0.11)	0.20 (0.15)			
	Micro-GES	0.11 (0.21)	0.44 (0.24)	0.65 (0.13)		
PB	BW	0.37 (0.11)	0.15 (0.14)	0.53 (0.12)	0.49 (0.20)	
	Micro-GES	0.22 (0.25)	0.27 (0.28)	0.29 (0.28)	0.01 (0.40)	− 0.14 (0.21)

### Ranking of EBV across subpopulations

**Fig. 1:**
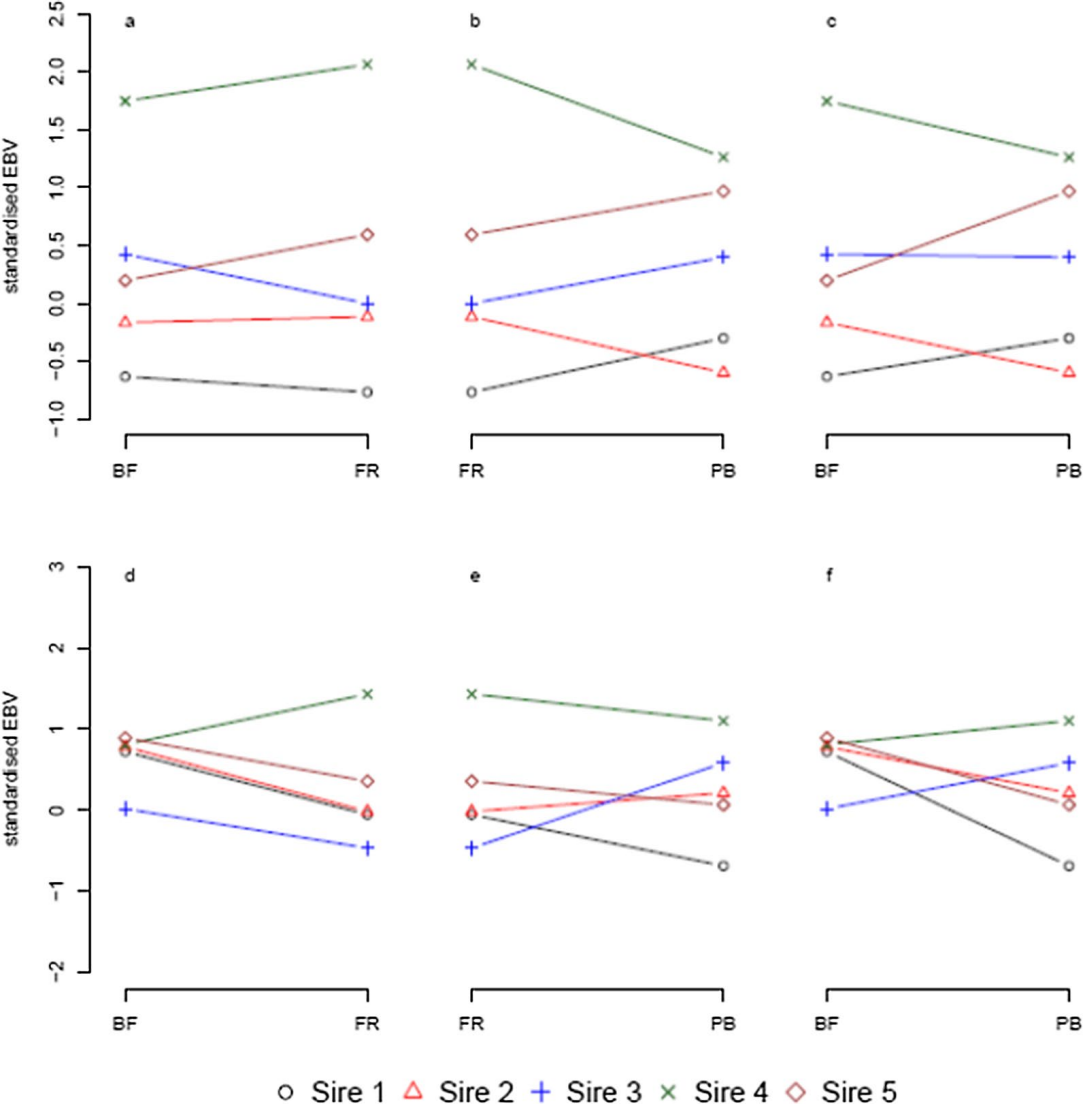
Ranking of the standardised EBV of the five sires with the most uniform distribution of offspring among the three subpopulations. **a**–**c** Standardised EBV for body weight. **d**–**f** Standardised EBV of micro-genetic environmental sensitivity (GES) of body weight. **a**, **d** Comparison between the crossbred in Burkina Faso (BF) and France (FR). **b**, **e** Comparison between FR and the purebred (PB). **c**, **f** Comparison between BF and PB

The EBV of the five sires with the least variation in the numbers of offspring across the three subpopulations are shown in Fig. 1.

Ranking of sires changed between subpopulations for both BW and micro-GES of BW. For the EBV of BW, re-ranking was more common when the EBV were based on records from the BF subpopulation compared to EBV based on records from the PB subpopulation, with four out of five sires ranking differently. The sire with the highest EBV for BW (sire 1, black circle) was the same for all three subpopulations.

For the EBV of micro-GES of BW, four out of five sires changed rank when the EBV were based on records from the PB subpopulation compared to EBV based on records from either of the crossbred subpopulations. Sire 2 (red triangle) had the same rank (third) for micro-GES for all three subpopulations.

## Discussion

### Micro-GES of BW within subpopulations

Significant micro-GES was found for BW in the current study, with the heritability estimates in line with those previously reported for micro-GES of BW in broiler chickens. Mulder et al. [11] reported heritability estimates on the measurement scale ranging from 0.034 to 0.047 for the dispersion for BW for female broilers of a purebred dam line analysed using a two-step variability approach, as well as estimates of the genetic coefficient of variation of micro-GES ranging from 0.44 to 0.57, which were considerably smaller than those estimated in the current study. Differences in heritability estimates between the current study and that of Mulder et al. [11] are unlikely to be caused by the difference in methods. While DHGLM have been found to result in greater accuracy of the EBV for micro-GES compared to a two-step variability model, the estimated variance components are generally similar when the two types of models are applied to the same dataset [21]. This indicates that the observed differences in estimates of variance components and heritabilities between the current study and that of Mulder et al. [11] may be largely due to differences in genetics and macro-environments. The records in the study by Mulder et al. [11] were obtained on chickens reared in both floor housing and cage housing, while the chickens in the current study were all reared in floor housing. Furthermore, while both studies used data provided by Hendrix Genetics, the birds were reared at different facilities with different management. Thus, it is possible that macro-environmental differences can explain some of the differences between the two studies, since it is shown here that micro-GES of BW depends on the macro-environment. However, it is still likely that differences in genetics contribute substantially to the differences in micro-GES of BW between the current study and that of Mulder et al. [11]. The S-line in the SAPPSA project is a slow-growing broiler line and PB had an average BW of 640 g (SD 117, Table 1) at 40–55 days of age. The female offspring of the dam line analysed by Mulder et al. [11] had an average BW of 2048 g (SD 217) at 43–53 days of age, which indicates that it was a faster growing broiler chicken line than the S-line. Estimates of the heritability of BW were also considerably different between the current study and that of Mulder et al. [11], suggesting that BW is a line-specific trait. It is therefore reasonable to expect micro-GES of BW to be line-specific as well.

### Micro-GES of BW across subpopulations

The results presented here show that sensitivity to micro-environments can be impacted by both macro-environmental and purebred-crossbred differences. There were considerable differences in estimates of both heritability and of the genetic coefficient of variation of micro-GES between BF and the other two subpopulations, with larger variances and higher heritabilities of micro-GES for BW in the BF subpopulation. The differences in heritability and variance estimates between the BF and PB subpopulations could be the result of both macro-environmental and purebred-crossbred differences. However, as the differences were generally larger between the BF and FR than between the FR and PB subpopulations, it is possible that macro-environmental differences were the primary cause of the observed differences in heritability and variances of micro-GES. Estimates of the heritability of micro-GES of BW in the FR and PB subpopulations were similar, further supporting the hypothesis that macro-environmental differences had a greater impact on micro-GES than purebred-crossbred differences. In a previous study on micro-GES of eggshell colour, minor differences in heritabilities of micro-GES were found between single-caged purebred chickens and family-caged crossbred chickens [12]. The minor differences in estimates of heritability of micro-GES of eggshell colour observed by Mulder et al. [12] were caused by both purebred-crossbred differences and macro-environmental differences due to the use of individual cages for the purebred chickens and family cages for the crossbred chickens. This suggests that the combination of purebred-crossbred differences and minor differences in housing does not cause substantial differences in scale of micro-GES in chicken. Thus, the scale differences observed in the current study are likely caused primarily by the large macro-environmental differences between countries.

Estimates of the genetic correlations for micro-GES of BW between the different subpopulations were low to moderate, suggesting that re-ranking of sires based on their EBV for micro-GES of BW across the subpopulations were expected, as shown in Fig. 1d–f. The estimated genetic correlation reported here were lower than the estimate of 0.70 (SE 0.19) reported by Mulder et al. [12] between eggshell colour in purebred laying hens reared in individual cages and crossbreds reared in family cages. In the current study, the macro-environmental differences were a combination of different management strategies and different climates between the facilities in Burkina Faso and France. Thus, the difference in estimates of genetic correlations between the current study and the study by Mulder et al. [12] could be due to larger differences between macro-environments in the current study. Alternatively, micro-GES of BW could be more responsive to purebred-crossbred and/or macro-environmental differences than micro-GES of eggshell colour. Both macro-environmental differences and purebred-crossbred differences, individually and combined, caused the re-ranking of EBV for micro-GES of BW observed in Fig. 1d–f, as shown by the moderately positive genetic correlation estimates between all subpopulations. The lowest genetic correlation estimate was observed between the BF and PB subpopulations, which indicates that purebred-crossbred and macro-environmental differences can amplify each other, thus causing more re-ranking of EBV than each effect individually.

While re-ranking based on EBV is expected to occur across all subpopulations, the genetic correlation estimates for micro-GES between different subpopulations were associated with relatively large SE (SE > 0.2), which means that the expected amount of re-ranking of EBV is uncertain. Mulder et al. [12] also reported relatively large SE for the estimate of the genetic correlation for micro-GES of eggshell colour between purebred and crossbred laying hens. Both the estimate and the SE of the estimate of the genetic correlations, and the accuracy of that estimate, for the same trait in different subpopulations depends on the genetic connectivity between the subpopulations, among other factors. In the current study, 66 sires had offspring in both the BF and FR subpopulations, 65 sires had offspring in both the PB and FR subpopulations, and 103 had offspring in both the BF and PB subpopulations. In the study by Mulder et al. [12], 71 sires had records for both purebred and crossbred laying hens. It is likely that the number of shared sires that are needed to obtain accurate estimates of genetic correlations for micro-GES between different subpopulations has to be larger. In a simulation study, Madsen et al. [22] estimated genetic correlations between a trait and the dispersion of another trait and reported SE of approximately 0.01 when the number of shared sires between the two traits was 200 (each sire had 100 offspring with a record for each trait). They did not examine the genetic correlation between micro-GES for different traits. Nevertheless, based on this, it is possible that the SE of genetic correlation estimates may be reduced as more records are collected in the SAPPSA project. Consequently, in the future, it may be possible to assess the amount of re-ranking of EBV more accurately.

### Opportunities for selection on micro-GES

A recurrent question regarding micro-GES is how much selection response is possible. To address this, Formoso-Rafferty et al. [7] conducted a selection experiment in which divergent lines of mice with high or low micro-GES of birth weight were successfully established. The population of mice had an initial heritability of dispersion of 0.008 and an initial genetic coefficient of variation of micro-GES of 0.22, respectively [7], both lower than observed in this study. However, the estimated variance of dispersion was only significantly different from zero for the BF and FR subpopulations. Thus, there is reason to believe that increasing uniformity of BW through selection could be obtained in the BF and FR subpopulations but not in the PB subpopulation.

It is important to note that selection experiments investigating micro-GES generally establish divergent lines through single-trait selection on micro-GES. Single-trait selection is not commonly practised in most breeding schemes. Instead, breeding animals are generally selected using selection indices for which traits are assigned weights depending on their relative importance for the breeding program. In a simulation study, Mulder et al. [10] showed that simultaneous improvement of the mean and micro-GES of a trait using index selection with a weight of 1 for the trait and − 1 for micro-GES of the trait in a sib testing scheme requires a heritability of dispersion of at least 0.02. Mulder et al. [10] also found that the selection differential for micro-GES decreased from − 0.118 to − 0.001 when the ratio between the additive genetic variance of micro-GES on the log-scale and the direct additive genetic variance of the trait decreased from 1 to 0.02. Our estimate of the heritability of dispersion was significantly higher than 0.02 in the BF and FR subpopulations, while the ratio of the additive genetic variance of micro-GES of BW to additive genetic variance of BW was estimated to be ≤ 0.0004. Based on these results, it is therefore uncertain that index selection on BW and micro-GES of BW would result in significant improvement in micro-GES of BW in the BF and FR subpopulations.

### Impacts of full-sibs on sire DHGLMs

The multi-trait DHGLM used to analyse BW and micro-GES of BW in the current study was implemented on a sire level, in part, because BW was not repeatedly measured on all birds and repeated records are necessary for accurate estimation of micro-GES using a DHGLM on the animal level [16], but also because no dam pedigree was available. The use of a sire model could have impacted the results because sire models assume that offspring are half-sibs, i.e., they have a genetic relationship of 0.25, and therefore that all genetic resemblance with a sire progeny group is due to the sire. Under this assumption, the estimated additive genetic sire variance is then ¼ of the additive genetic variance [3]. The assumption that all genetic resemblance between a sire’s progeny is due to the sire does not hold in datasets that include a mix of full- and half-sibs, because in such data the genetic relationships among a sire’s progeny group deviates from 0.25. To assess the impact of having a mix of full- and half-sibs, we conducted an additional small simulation study (see Additional file 1—simulation study with Tables S1 and S2) that showed that the sire variance of both the mean value of the trait and of micro-GES was overestimated when data contained both full- and half-sibs, but that it was accurately estimated when data contained only half-sibs. The estimate of the additive genetic variance of the mean on the animal level was less biased when the relationship within sire progeny groups was used in the conversion from sire-level to animal-level variances instead of the assumed 0.25 relationship, although this did not completely remove the bias of the estimated sire variance of micro-GES. It is not possible to say how much the estimates of the additive genetic variances in the real data are biased, since we could not calculate the actual relationship among the progeny of each sire from the available data. Thus, the estimates presented here remain the best estimates available.

## Conclusions

This study examined micro-GES of BW in three subpopulations of chicken. One subpopulation consisted of purebred females of a slow-growing broiler line reared in France. Two subpopulations consisted of crossbred female chicken with the same slow-growing broiler as sire line, reared either in France or Burkina Faso. The results show that micro-GES existed in the crossbred chicken reared in Burkina Faso with low heritabilities and high genetic coefficients of variation. Based on these results, it should be possible to increase the uniformity of BW in the examined subpopulations by selecting for reduced micro-GES in the purebred line used as sire line for the crossbred subpopulations. The results also show that micro-GES of BW exhibited both heterogeneity of variances and re-ranking of EBV due to macro-environmental differences and due to purebred-crossbred interactions. These findings highlight the need for investigating micro-GES in all subpopulations included in a selection program.

### Supplementary Information

**Additional file 1.** Simulation study of the impacts of mixed full- and half-sib data in sire models. Methods used to conduct the simulation study examining the impact of having mixed full- and half-sib data in sire models and the results of this simulation study in Tables S1 and S2. **Table S1.** Estimated additive genetic variance from the analysis of simulated datasets with varying mating ratio (standard error of the mean across 100 replicates) on the level of the model and converted to the animal level assuming the additive genetic sire variance is 0.25 of the additive genetic variance or using the average relationship of sire progeny groups. **Table S2.** Estimated additive genetic variance of micro-GES from the analysis of simulated datasets with varying mating ratio (standard error of the mean across 100 replicates) on the level of the model and converted to the animal level assuming the additive genetic sire variance is 0.25 of the additive genetic variance or using the actual relationship of sire progeny groups.

## Data Availability

Any data request should be made directly to Hendrix Genetics.
