# Supplementary material for: Micro-genetic environmental sensitivity across macro-environments of chickens reared in Burkina Faso and France
Source: Genet Sel Evol. 2023 Nov 30;55:85. doi: 10.1186/s12711-023-00854-7 (PMC10688495; doi:10.1186/s12711-023-00854-7)
Supplement: Supplementary file 1 — Additional file 1. Simulation study of the impacts of mixed full- and half-sib data in sire models. Methods used to conduct the simulation study examining the impact of having mixed full- and half-sib data in sire models and the results of this simulation study in Tables S1 and S2. Table S1. Estimated additive genetic variance from the analysis of simulated datasets with varying mating ratio (standard error of the mean across 100 replicates) on the level of the model and converted to the animal level assuming the additive genetic sire variance is 0.25 of the additive genetic variance or using the average relationship of sire progeny groups. Table S2. Estimated additive genetic variance of micro-GES from the analysis of simulated datasets with varying mating ratio (standard error of the mean across 100 replicates) on the level of the model and converted to the animal level assuming the additive genetic sire variance is 0.25 of the additive genetic variance or using the actual relationship of sire progeny groups. [file 12711_2023_854_MOESM1_ESM.docx]

## Simulation study of the impacts of mixed full- and half-sib data in sire models

**Methods**

In this study, a sire DHGLM was applied to a dataset with a mixture of full- and half-sibs without dam information. To investigate the impacts of using sire models and sire DHGLMs when analysing data with a mixture of full- and half-sibs a simulations study was conducted where 200 sires were mated to either 10 or 18 dams and each mating produced 5 offspring. Sires and dams were unrelated, and mating was random. Breeding values for the mean ($a$) and micro-GES ($a_{d}$) were simulated following

| $N\left( \left[ \begin{matrix} 0 \\ 0 \end{matrix} \right]\boldsymbol{,}\left[ \begin{matrix} 2000 & 0 \\ 0 & 1 \end{matrix} \right]\otimes\mathbf{A} \right)$ | (17) |
| --- | --- |

where **A** is the pedigree based additive genetic relationship matrix. The residual variance ($\sigma_{e}^{2}$) was 2000 and the population mean ($\mu$) was 100. Phenotypes were calculated for the progeny as

| $y_{i}=\mu+a_{i}+\exp\left( 0.5\ln\left( \sigma_{e}^{2} \right)+0.5{a_{d}}_{i} \right)\varepsilon_{i}$ | (18) |
| --- | --- |

where $\varepsilon_{i}$ was a random environmental variable drawn from $N\left( 0,1 \right)$ [16].

The true variances were chosen based on the results from BF.

In the scenario where sires were mated to 10 dams, phenotypes were simulated for 5000 offspring, and all were used for analysis. When the mating ratio was 18 dams per sire, 9000 phenotypes were simulated and used for analysis.

The simulated records were analysed using a traditional animal model, a traditional sire model and a sire DHGLM with only the additive genetic effects as random effects and the population mean as fixed effects.

A dataset with only half-sibs was also simulated, where each sire was mated to 90 dams and each mating produced one offspring. The remaining parameters were as for the simulations with both full- and half-sibs. The half-sib dataset was analysed using the same models as the mixed full- and half-sib datasets.

Each simulation was replicated 100 times. Within each replicate the estimated variance components were saved for all models and, for the sire model and DHGLM. The variance components were converted from sire to animal level using equation (6) and (8), either by assuming that all sires’ progeny have a relationship of 0.25, or using the actual relationship among sires’ progeny instead. The mean and standard error of the mean where then calculated across all replicates and reported in the results section.

**Results**

The simulation study showed that the additive genetic variance of both the trait itself and micro-GES was overestimated when the data contained a mix of full- and half-sibs and the additive genetic variance was calculated as 4 times the additive genetic sire variance (Tables S1 and S2). In the scenario where the mating ratio was 1:1 (no full-sibs) all additive genetic variances were accurately estimated.

For the scenario where the mating ratio was 1:10 (highest proportion of full sibs per progeny group) the additive genetic variance was significantly overestimated by 9 and 10% for the sire model and DHGLM, respectively, when assuming that the estimated sire variance was 0.25 of the additive genetic variance. For the scenario where the mating ratio was 1:18, the additive genetic variance was significantly overestimated by 6% and 7% in the sire model and DHGLM, respectively. Using the actual relationship among sires’ progeny instead of 0.25, resulted in additive genetic variances that were not significantly different from the true additive genetic variance, regardless of model and mating ratio.

**Table S1 Estimated additive genetic variance from analysis of simulated datasets with varying mating ratio (standard error of the mean across 100 replicates) on the level of the model and converted to the animal level assuming the additive genetic sire variance is 0.25 of the additive genetic variance or using the average relationship of sire progeny groups.**

| **Mating ratio** | **Model** | **Estimated**  **genetic variance** | **^­­^Additive genetic variance^+^** | | |
| --- | --- | --- | --- | --- | --- |
|  |  |  | **0.25** | **aR** | |
| 1:1 | Animal | 1975.53 (21.05) | - | - |  |
|  | Sire | 493.88 (5.26) | 1975.53 (21.05) | 1975.53 (21.05) |  |
|  | DHGLM | 495.69 (5.24 | 1982.75 (20.98) | 1982.75 (20.98) |  |
| 1:10 | Animal | 2014.07 (18.03) | - | - |  |
|  | Sire | 545.23 (7.14) | 2180.92 (28.56) | 1982.65 (25.97) |  |
|  | DHGLM | 547.56 (7.08) | 2190.22 (28.33) | 1991.11 (25.75) |  |
| 1:18 | Animal | 2013.26 (10.91) | - | - |  |
|  | Sire | 531.85 (5.65) | 2127.39 (22.61) | 2015.42 (21.42) |  |
|  | DHGLM | 269.96 (4.88) | 2015.42 (21.42) | 2020.06 (21.44) |  |

^+^true additive genetic variance of trait= 2000, aR = actual relationship of sire progeny groups

The DHGLM overestimated the additive genetic variance due to micro-GES by 39 and 25% when the mating ratio was 1:10 and 1:18, respectively (Table 5). This overestimation reduced to 12 and 11%, but remained significant, when using the actual sire progeny group relationship instead of 0.25.

**Table S2 Estimated additive genetic variance of micro-GES from analysis of simulated datasets with varying mating ratio (standard error of the mean across 100 replicates) on the level of the model and converted to the animal level assuming the additive genetic sire variance is 0.25 of the additive genetic variance or using the actual relationship of sire progeny groups.**

| **Mating ratio** | **Model** | **Estimated**  **genetic variance** | **^­­^Additive genetic variance^+^** | |
| --- | --- | --- | --- | --- |
|  |  |  | **0.25** | **aR** |
| 1:1 | DHGLM | 0.11 (0.00) | 1.03 (0.02) | 1.03 (0.02) |
| 1:10 | DHGLM | 0.12 (0.00) | 1.39 (0.03) | 1.12 (0.03) |
| 1:18 | DHGLM | 0.12 (0.00) | 1.25 (0.02) | 1.11 (0.02) |

**^+^**true additive genetic variance of micro-GES=1, aR = actual relationship of sire progeny groups
